# Morgan's Plastic Gold

**Published:** 1867-10

**Authors:** 


*Morgan's Plastic Gold.*—The success which has attended our use of this new form of gold for filling teeth, and the favorable opinions expressed concerning it by excellent operators, warrants us in recommending it to our readers for trial. The following directions are given for its use.

"This preparation is to be manipulated with instruments of shallow serration, and requires some little practice to become familiar with its working properties. It may be used in quite large pieces without fear of leaving a porous sub-surface from superficial consolidation. Although very *cohesive*, care must be exercised in first carrying it into the cavity to work the material *gently* together, after which it may be forcibly condensed, subsequently admitting of pressure with the finest points without danger of cutting or flaking the surface, which equals in density and toughness the finest foil."

